# A Design to Investigate the Feasibility and Effects of Partnered Ballroom Dancing on People With Parkinson Disease: Randomized Controlled Trial Protocol

**DOI:** 10.2196/resprot.3184

**Published:** 2014-07-22

**Authors:** Ann Ashburn, Lisa Roberts, Ruth Pickering, Helen Clare Roberts, Rose Wiles, Dorit Kunkel, Sophia Hulbert, Judy Robison, Carolyn Fitton

**Affiliations:** ^1^University of SouthamptonFaculty of Health SciencesUniversity of SouthamptonSouthamptonUnited Kingdom; ^2^University of SouthamptonFaculty of MedicineUniversity of SouthamptonSouthamptonUnited Kingdom; ^3^Academic Geriatric MedicineFaculty of MedicineUniversity of SouthamptonSouthamptonUnited Kingdom; ^4^University of SouthamptonFaculty of Social SciencesUniversity of SouthamptonSouthamptonUnited Kingdom

**Keywords:** Parkinson disease, ballroom dancing, balance, posture

## Abstract

**Background:**

Self-help and physical leisure activities has become increasingly important in the maintenance of safe and functional mobility among an increasingly elderly population. Preventing the cycle of deterioration, falling, inactivity, dependency, and secondary complications in people with Parkinson disease (PD) is a priority. Research has shown that people with PD are interested in dance and although the few existing trials are small, initial proof of principle trials from the United States have demonstrated beneficial effects on balance control, gait, and activity levels. To our knowledge, there has been no research into long-term effects, cost effectiveness, the influence on spinal posture and turning, or the personal insights of dance participants.

**Objective:**

The purpose of this study was to determine the methodological feasibility of conducting a definitive phase III trial to evaluate the benefits of dance in people with PD. We will build on the proof of principle trials by addressing gaps in knowledge, focusing on areas of greatest methodological uncertainty; the choice of dances and intensity of the program; for the main trial, the availability of partners, the suitability of the currently envisaged primary outcomes, balance and spinal posture; and the key costs of delivering and participating in a dance program to inform economic evaluation.

**Methods:**

Fifty participants (mild-to-moderate condition) will be randomized to the control (usual care) or experimental (dance plus usual care) groups at a ratio of 15:35. Dance will be taught by professional teachers in a dance center in the South of England. Each participant in the experimental group will dance with his or her spouse, a friend, or a partner from a bank of volunteers. A blinded assessor will complete clinical measures and self-reported ability at baseline, and at 3 and 6 months after randomization. A qualitative study of a subgroup of participants and partners will examine user’s views about the appropriateness and acceptability of the intervention, assessment protocol, and general trial procedures. Procedures for an economic evaluation of dance for health care will be developed for the main trial.

**Results:**

Recruitment began in January 2013 and the last participant is expected to complete the trial follow-up in June 2014.

**Conclusions:**

Findings from our study may provide novel insights into the way people with PD become involved in dance, their views and opinions, and the suitability of our primary and secondary outcomes.

**Trial Registration:**

International Standard Randomized Controlled Trial Number (ISRCTN): 63088686; http://www.controlled-trials.com/ISRCTN63088686/63088686 (Archived by WebCite at http://www.webcitation.org/6QYyjehP7).

## Introduction

Parkinson disease (PD) is a common, progressive neurological condition estimated to affect 100-180/100,000 of the population [1]. People with this condition frequently experience deterioration of their spinal posture, mobility, and stability, leading to dependency and falls; therefore, preventing the cycle of inactivity and secondary complications is a priority. Despite disabling movement deficits, access to physiotherapy is limited [2]. Poor performance on measures of balance and functional tasks is common and has been associated with generalized deficits of attention and fall events, leading to decreased quality of life among sufferers [3]. The problem of unwanted secondary deterioration is of considerable concern.

People with PD experience slow movements (bradykinesia), rigidity, resting tremors, and abnormalities of postural reflexes. Gait characteristics include a shuffling pattern of walking with increased flexion of the hips and thoracic spine and reduced movement at the ankle with loss of heel strike. Restricted rotational movements of the head and trunk can contribute to the overall instability experienced by individuals. Key approaches to improving the function and activity of people with PD include balance and strengthening exercises [4] as well as strategies using rhythmical cueing to enable people to initiate movements [5]. Ballroom dancing comprises many of these facilitating components described; hence, the reason that dance was considered an appropriate activity for people with PD. The music provides the rhythmical cue for stimulating movement and the stepping and turning activities challenge balance control.

Self-help and physical leisure activities are increasingly important in the battle to maintain safe functional mobility among people with PD. Although there is growing evidence of the benefits of exercise for people with PD, research into the benefits of long-term exercises through leisure pursuits such as dance and self-help activities is limited. Research has shown that people with PD are interested in dance and early findings suggest a positive effect on balance and gait following Argentine tango classes, partnered or nonpartnered [6]. The effectiveness literature on dance in PD is growing, although the findings of the early studies have been limited by small sample sizes—the average study recruiting under 20 participants, and the largest recruited 52. These studies have shown proof of principle that dance, usually Argentine tango, for people with PD can be delivered [6-12] and can positively influence balance control and gait. Another study compared the tango with the waltz and the foxtrot and dance was superior to no dance but there was no distinction among dances. One-hour sessions, twice weekly for 10 weeks is beneficial. Only one recently completed study has evaluated dance delivered in the community. Earhart and Duncan [13] and Foster and Earhart [14] described a 12-month community-based tango program that demonstrated that dancing using community facilities was possible and they showed that those participants who danced increased their participation in activities of daily living. Positive effects were also shown when people were tested off medication.

Gaps in knowledge have been highlighted by a number of researchers. Two meta-analyses have identified the need for more well-designed randomized controlled trials and qualitative studies of the dance experience of people with PD [15,16]. Hackney and Earhart [6] highlighted a gap of knowledge concerning the long-term effects and cost effectiveness of dance. No existing studies have examined the influence of dance on spinal posture and turning (a task that inherently makes people unstable) or the personal insights of participants about their experiences with dance. We were aware that no-one had examined the feasibility of teaching a number of standard ballroom dances to people with PD through a local dance center in the UK or how a center would cope with finding dance partners for people with PD, and it is important to explore these feasibility issues before running a large multicenter trial.

Because this is a feasibility study, we will not be testing a hypothesis but will determine the methodological feasibility of evaluating the benefits of multiple dances taught through a dance center as a precursor to a definitive phase III trial. We need to understand how much dance people with PD and healthy partners will tolerate, which dances work best, how easy it is for dance teachers and dance centers to accommodate the needs of people with PD, and identify barriers and facilitators to participation in dance including the financial costs. We will build on the proof of principle studies [6-8] from the United States, address gaps in knowledge in planning a definitive trial of the impact of dance on balance and spinal posture, as well as turning, long-term follow up, economic evaluation, and user views. We will examine the appropriateness of primary and secondary outcomes currently envisaged for the future main trial and provide novel insight into user views on involvement in dance through a qualitative component, and develop procedures for economic evaluation for a future trial.

Specific questions of the feasibility study are:

1. What are the key routes to successful recruitment of healthy partners, people with PD, and dance centers to a randomized controlled trial of dance?

2. What are the most appropriate dances for people with PD?

3. How frequent, and how much dance is reasonable and likely to result in benefit for people with PD and healthy partners?

4. What specific adjustments do dance centers need to make regarding the delivery of dance to this group of people?

5. What level of dancing will be sustained to 3 and 6 months?

6. Will we obtain high quality data on spinal posture, balance, turning, and walking during home assessments?

7. Will we obtain high quality data on self-reported health status and balance confidence?

8. How appropriate and burdensome is the battery of assessments?

## Methods

### Overview

**Figure 1:**
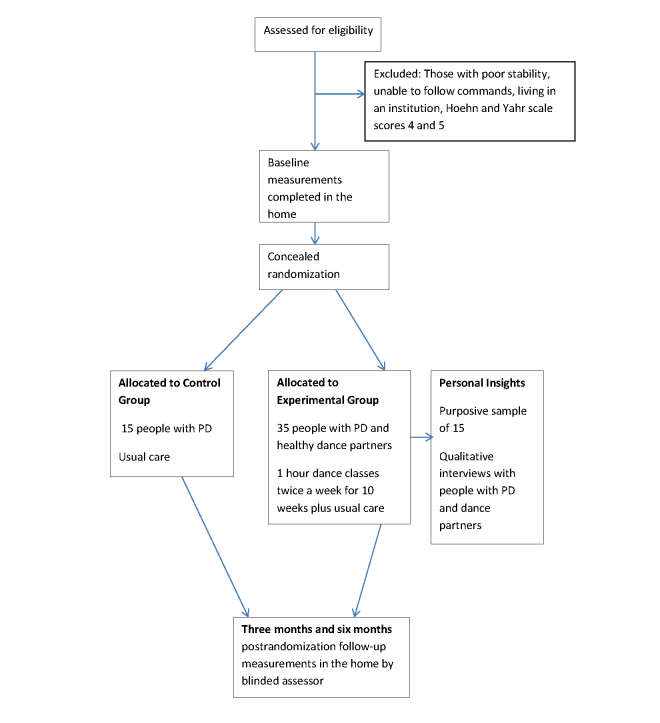
Flowchart of trial design.

We followed the Standard Protocol Items: Recommendations for Interventional Trials (SPIRIT) guidelines [17] for developing the protocol and Figure 1 presents a diagrammatic representation of the study based on the Consolidated Standards of Reporting Trials (CONSORT) [18] flowchart. Two research fellows, physiotherapists with experience in PD, will conduct the research trial in collaboration with the dance teachers in a dance center in the South of England. Research fellow A will recruit participants and conduct baseline and follow-up assessments and will be blind to group allocation. Once a participant has consented to the trial, their name will be passed on to the second research fellow B, who will carry out the random allocation procedures, be present throughout all the dance classes, help arrange transport to the dance classes according to need, and organize volunteers for dance partners.

### Participants With Parkinson Disease

Research fellow A will obtain informed consent and recruit 50 people with PD from outpatient services, local support groups (Parkinson’s UK), and the DeNDRoN (Dementia and Neurodegenerative Disease Research Network, a clinical research network for dementia and neurodegenerative conditions). Eligible people with PD will have a confirmed diagnosis of PD, Hoehn and Yahr [19] scale of 1-3 indicating mild-to-moderate mobility and stability; will be able to stand, turn, and walk safely and unsupported but may experience freezing of gait [20]; will live at home; understand and follow commands, through a screen for cognitive impairment [21]; and be able to tolerate the dance intervention. People with PD lacking sufficient stability to dance with another person in the clinical opinion of research fellow A (eg, in danger of losing balance control each time they turn around) will be excluded. The decision as to whether a potential participant with limited mobility can safely undertake the dance intervention will be based primarily on their performance on the Berg balance scale (BBS) [22] and the motor component of the unified Parkinson’s disease rating score (UPDRS) [23]. For example, someone scoring 0 on questions 7, 8, or 13 of the BBS, indicating inability to “stand unsupported with feet together,” inability “to attempt to reach forward” in standing without losing balance or inability “to stand unsupported with one foot in front of the other” will be considered ineligible. The UPDRS motor component gait questions 29 and 30 will also be used as indicators: a severe disturbance of gait that requires constant assistance or lack of postural stability to such an extent that balance is spontaneously lost in standing, will also discount potential participants.

### Healthy Dance Partners

Healthy people identified by participants in the dance group will act as dance partners. Some people with PD will be single; others may have a spouse unwilling to participate, so relatives, volunteers, or responders to an advert will also be considered. The feasibility of recruiting healthy dance partners will be examined in the study. Our inclusion criteria for the dance partners are similar in age to people with PD, able to understand and follow commands, willing to participate, and able to tolerate the dance intervention. Evidence of a neurological condition, vestibular impairment, or multiple falls would lead to exclusion, but experience of a single fall would be acceptable (risk of falling is linked to repeated falling, not a single fall event) [24]. Otherwise healthy individuals who are considered to be at risk of instability while dancing will be excluded.

### Randomization

After the baseline assessment, eligible and consenting people with PD will be randomized to the dance or control group. Research fellow B obtained allocations by telephone from the trial medical statistician. Allocation will be randomized in 1 block of 11 participants (8 dance and 3 control), and 3 blocks of 13 (9 dance and 4 control), to yield participants entering each of 4 consecutive series of dance classes. The feasibility trial is too small to incorporate stratification. The dance-to-control ratio of 35:15 was chosen to maximize the number of people with PD experiencing the dance classes, while still including some control participants for feedback on the acceptability of randomization to control, retention, and fidelity in this group. The research fellow will contact participants to inform them of their allocation and of the times of their dance classes.

### Interventions

#### Control Group

Participants in this group will continue with usual care. Usual care comprises medication, attendance at medical clinics, and review by PD nurses. Exercise therapy may be accessed but this is not routinely prescribed in the UK. As a way of encouraging adherence to follow-up at the end of the trial, participants in the control group will be offered vouchers to attend nontrial dance classes at the dance center.

#### Dance Group

**Figure 2:**
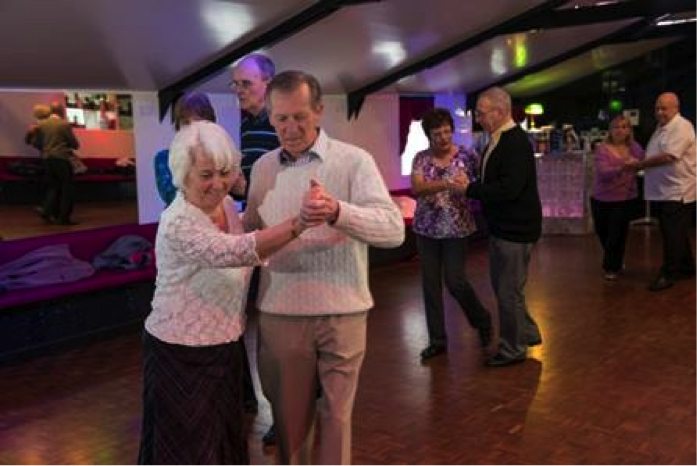
Participants enjoying a dance class.

In addition to usual care, participants will attend dance classes (with a healthy partner) at a local dance center, led by a professional dance teacher and supported by a second teacher and research fellow B (Figure 2). Classes will last 1 hour, twice a week for 10 weeks. The length and number of sessions was chosen following discussions with the dance teacher and based on results from published literature [6]. Care will be taken to ensure that the pace of teaching is appropriate and will reflect that recommended by Hackney and Earhart [6]. Class sizes will be small and supervision from both the dance teachers and research fellow B will minimize any risk of falling. Participants will learn 6 dances (3 ballroom and 3 Latin American dances). Each session will start with a warm-up, then new steps will be introduced and practiced, and a record of the session will be kept. Proposed dances included the social foxtrot, the waltz, the cha cha, ballroom tango, the rumba, and rock and roll. Emphasis will be placed on encouraging individuals to extend their postures, turn their heads and trunks, heel strikes, and toe push-offs because these movements are affected by PD and associated with changes in balance responses. The dance program will be tailored to individual capabilities in each class (the dance teachers will dance with couples who were struggling), and the research fellow will ensure the safety and well-being of participants and their partners. The research fellow will keep a record of each class, and some of the classes will be videotaped (with consent from participants).

### Assessments

Research fellow A will carry out baseline and follow-up assessments at 3 and 6 months and will be blinded to participant group allocation. Prior to each assessment, participants will be reminded not to reveal whether they have been dancing, and following each assessment the assessor will be asked to record awareness of group allocation. The assessments will be organized at similar times of the day, will last approximately 1 hour, and be completed in the person’s home approximately mid-point in the medication cycle.

Data collected for the anticipated primary outcome measures for the main trial will be the BBS [22], a categorical scale of balance activities (a high score is good with a maximum of 56), and spinal posture, which will be measured using the spinal mouse [25]. The spinal mouse is a handheld device that can measure the position and mobility of the pelvis, and lumbar and thoracic spine by recording the segmental angles as it is rolled over the spine. This device has been used with people with PD [26] and the data will describe angles of the participants’ spinal segments in the standing position and the overall degree of forward inclination in relation to upright. We used these tests in previous research studies [27].

At baseline, we will characterize our sample using the following measures: demographic data; measures of disease severity (Hoehn and Yahr Scale [19]; 1-5, high score is poor) and UPDRS motor section [23] (0-56, high score is poor); medication (using levodopa equivalent daily dose); freezing of gait questionnaire [20] (0-24, high score is poor); the Montreal cognitive assessment of cognitive function [21] (0-30, high score is good); and we will ask participants to retrospectively recall fall events during the previous 12 months using a standardized questionnaire [28].

Outcome measures will be recorded at baseline, and at 3 and 6 months and included the primary outcome measures, the Berg balance scale [22] and the spinal mouse [25]. The secondary outcomes include a measure of turning, the standing start 180° (SS180°) test in which the individual turns in both directions and the number of steps, time, and quality of turn are rated [29]; the timed up and go test, stand up from a chair and walk forward 3 meters and turn and return [30]; the PDQ39 (a self-completing questionnaire rating how hard it is to complete a range of everyday activities [31]; 0-100, low score indicates better health); the ABC, which is a questionnaire about balance confidence [32] (0-100, high score is good) ;the phone-FITT, a questionnaire delivered over the phone and designed to gather information about levels of the physical activity of older adults, such as how many times a week do you do housework activities such as tidying or dusting, walking, swimming [33], scoring is according to frequency of activities, duration, and intensity; and the Euroquol-5D, which is a simple quality-of-life measure [34] (1-5, high score is poor but a high visual analoge score is good).

At the final assessment, participants will be given a questionnaire about resource use that will be used in economic evaluation such as travel, refreshments, and buying shoes for the dance class and their views on the acceptability and benefits of the research project and to collate additional information needed for the economic evaluation. Participants will be asked to complete this questionnaire on their own time and to return it to the research team in the freepost envelope provided. We plan to ensure that the measurement battery is appropriate for use in a definitive multicenter phase III trial and not burdensome for the assessor or participants.

### Statistical Analysis

Feasibility issues will be addressed by examining numbers and percentages successfully recruited and completing various components of the trial protocol. The choice of outcomes for the main trial will be addressed by examining the completeness of data collection, relevance to issues raised as important by users, and comparison to related studies in the literature. The statistical analysis plan for the primary efficacy outcome in the main trial is currently envisaged to contrast the dancing and control groups (presented with 95% CI), after controlling for the primary outcome measured at baseline and center, either performed separately at 3, 6, and 12 months in analysis of covariance, or in a mixed model including both follow-up points. Depending on the primary outcome chosen, the corresponding modelling approach in logistic or Poisson models may be appropriate. Comparisons of outcome will be done on an intention-to-treat basis, with sensitivity analyses restricted to those completing the allocated interventions. The analysis of outcome data from the pilot randomized controlled trial, along with information from the literature, will inform the power calculation for the main trial. The current trial is not powered to demonstrate efficacy. We will examine the predictive power of baseline characteristics such as the Hoehn and Yahr severity, freezing of gait, and phone-FITT scores, to assess the benefit of additionally including them as stratifiers in the randomization of the main trial.

### Qualitative Substudy

#### Aim

The aim of the qualitative substudy will be to identify and explore the views of people with PD and their dance partners about the appropriateness and acceptability of the dance programs and the perceived impact on their mobility. Their views of the trial procedures will also be explored. An experienced qualitative researcher has been employed to complete this part of the study.

#### Methods

Fifteen of the 35 couples in the experimental group will be recruited to the qualitative study. Couples will be recruited with the aim of attaining maximum variation in the qualitative sample of the factors that may affect their experiences of the dance intervention, such as age, sex, and relationship with the dance partner.

#### Interviews

In-depth, semistructured, qualitative interviews will be conducted using an interview guide developed for the study. The interviews will be conducted in the home separately with the people with PD and their dance partners where possible (where couples are spouses this may not be possible) within 1 month of completing the dance program. With participants’ consent, the interviews will be audiorecorded and fully transcribed. For people with PD, the interviews will explore the following issues: their perceptions of the impact of PD on their day-to-day lives and their experience of physiotherapy or any other interventions since diagnosis that were designed to promote activity; and experiences with exercise activity (dance or other exercise activity) before their diagnosis of PD. The following issues will be explored with people with PD and their dance partners: their reasons for deciding to take part in the dance program and their perceptions and expectations of it, the number of sessions they attended and reasons for missing any sessions or dropping out, what it was like to take part, enjoyment of the activity, maintaining enthusiasm for the activity, the implications of working with a partner, perceptions of the impact of participating in the dance program, interest in continuing with dance classes or other activity, and their perceived impact on their mobility. Participants will also be asked about any personal costs they experienced while participating.

#### Analysis

Facilitated by QSR International’s NVivo 9.2 software, the data will be managed using Framework [35] and analyzed thematically to explore participants’ views about the acceptability and appropriateness of the dance program. Features of grounded theorizing and constant comparison will be used to identify and develop themes [36]. Data analysis will be undertaken by experienced social science qualitative researchers (JR and RW).

The framework is a staged approach that is well-suited to applied health research. In the first stage, the researchers will identify topics for an initial analytic framework based on prior understanding of the issues and concepts arising from close reading of the transcripts. In the second stage, participants’ accounts will be condensed on a case-by-case basis into charts according to the framework topics. The third stage will involve working through the data in detail to draw out themes or categories of experience that capture the full range of perspectives identifying commonalities and differences within and between participants.

The qualitative findings will be used to inform interpretation of the findings of the trial. Analysis of the qualitative data will take part in isolation from the trial researchers. Data from the qualitative study will be integrated at the end of the trial to avoid contamination.

Finally we will explore the views of those who choose not to take part in the study. Typically it is viewed as unethical or unnecessary to ask people to give reasons for declining to take part in research; however, in this study the information that is handed out as part of the recruitment process will give people the option to tell us if and why they find the dance intervention unattractive by completing an anonymous short questionnaire.

### Economic Evaluation Substudy

#### Aim

Our aim is to inform a future economic evaluation for the Phase III trial. We will use the EQ5D to assess quality of life.

#### Methods

A questionnaire about resource use such as travel, refreshments, and clothing (dance shoes) costs will be completed at 6 months. This information will enable us to judge the feasibility of this type of measure for people with PD. We will also calculate the resources needed to provide the intervention such as cost of dance lessons, hire of a hall, and personal expenditures.

## Results

Recruitment began in January 2013 and the last participant is expected to complete the trial follow-up in June 2014.

## Discussion

The main aim of this study is to determine the methodological feasibility of conducting a definitive phase III trial to evaluate the benefits of dance in people with PD. Recruitment to the feasibility trial began in January 2013 and the last participant is expected to complete their follow-up assessment in June 2014. Findings from our study will provide a novel insight into the way people with PD become involved in dance, their views and opinions, and the suitability of our primary and secondary outcomes. We will report on the challenges of recruiting healthy dance partners as well as people with PD, and because we believe the dance partnership is key to the success of the dance experience, we look forward to understanding better the ingredients required for success. We suspect that threats to the viability of the feasibility trial are likely to come from poor recruitment of healthy partners to dance with people with PD, demands and difficulties related to traveling to the dance center, and reluctance of people with PD to dance twice a week for 10 weeks. We anticipate that ease of travel and access to facilities such as the availability of car parking and taxis as well as environmental hazards will affect the experience. We will consider the steps that need to be taken to ensure safety and we are keen to analyze the types of dances that are enjoyed and those that create challenges for people with PD. We look forward to evaluating the potential benefits and challenges of running the dance classes for people with PD as well as providing practical information, such as participants’ preferences and experiences of the different dances, which has not previously been reported. With this study, we will be well placed to inform future research in this field.

## Abbreviations

BBS: Berg balance scale
CONSORT: Consolidated Standards of Reporting Trials
PD: Parkinson disease
SPIRIT: Standard Protocol Items: Recommendations for Interventional Trials
UPDRS: unified Parkinson’s disease rating score
